# In vitro culture of *Echinococcus multilocularis* producing protoscoleces and mouse infection with the cultured vesicles

**DOI:** 10.1186/s13071-016-1687-y

**Published:** 2016-07-25

**Authors:** Hui Wang, Jun Li, Baoping Guo, Li Zhao, Zhuangzhi Zhang, Donald P. McManus, Hao Wen, Wenbao Zhang

**Affiliations:** 1 State Key Laboratory Incubation Base of Xinjiang Major Diseases Research, Clinical Medical Research Institute, The First Affiliated Hospital of Xinjiang Medical University, Urumqi, Xinjiang 830054 China; 2 Veterinary Research Institute, Xinjiang Academy of Animal Science, Urumqi, Xinjiang 830000 China; 3 Molecular Parasitology Laboratory, Infectious Diseases Division, QIMR Berghofer Medical Research Institute, Brisbane, QLD Australia

**Keywords:** *Echinococcus multilocularis*, Protoscoleces, Vesicles, In vitro culture, Animal model

## Abstract

**Background:**

Alveolar echinococcosis (AE) is a lethal zoonosis caused by the fox-tapeworm *Echinococcus multilocularis*. The disease is difficult to treat and an effective therapeutic drug is urgently needed. Reliable models are essential for drug development. In this study, we developed both in vitro and in vivo models of larval *E. multilocularis*.

**Results:**

The protoscoleces (PSC) of *E. multilocularis* from jirds were successfully cultured in a modified RPMI1640 based medium containing 25 % (v/v) fetal bovine serum (FBS). After 100 days of culture, PSC developed to larval vesicles (small unilocular cysts) and the fast growing vesicles produced PSC in brood capsules. In addition, mice were intraperitoneally injected with 30 cultured small vesicles and 100 % of the mice had resulting metacestode masses.

**Conclusions:**

Larval protoscoleces and vesicles of *E. multilocularis* grow healthily in vitro in the RPMI1640 based medium containing 25 % FBS. *Echinococcus multilocularis in vitro* and in vivo models provide a valuable platform for investigating the biology of the parasite and screening effective therapeutic drugs against AE.

## Background

Alveolar echinococcosis (AE) is one of the most lethal helminthic infections in humans [1]. The disease is caused by the fox-tapeworm *Echinococcus multilocularis*, which occurs in most areas of the northern hemisphere [2, 3]. Recent epidemiological studies showed that AE is highly endemic in Central Asia, including western China, Kazakhstan and Kyrgyzstan [4, 5]. AE is a chronic parasitic disease and can remain asymptomatic in a patient for up to 15 years [6]. When an AE patient feels ill and requests to see a doctor, it is normally at a late stage. In this late stage, the parasite causes liver damage through its production of an infiltrating structure consisting of numerous small vesicles embedded in stroma of connective tissue, which makes surgical resection difficult and secondary re-infection often occurs [7]. Normally, surgical removal, if applicable, is the only curative method for treating AE and patients have to take albendazole (ABZ) and/or mebendazole (MBZ) after surgical treatment for at least 2 years [8]. Inoperable AE patients must undergo long term, often life-long, chemotherapy with ABZ and/or MBZ [9, 10]. Recent study has shown that these drugs are not parasiticidal in vivo against AE, and only inhibit *E. multilocularis* growth. This is the reason that AE patients have to take the drugs for a long time [11]. In addition, the occurrence of side effects with these drugs has been often reported, leading to discontinuation of treatment or to progressive disease. An effective therapeutic drug for AE is urgently needed.

Metacestodes of *E. multilocularis* are fluid-filled grape-like vesicles. The asexually proliferating vesicles cause the disease and the vesicles have been used for drug screening [4, 12, 13]. A simple and stable method for culturing the metacestodes of the parasite will be beneficial not only for biological and physiological studies, but also for speeding up drug development against AE. Several previous studies have achieved successful cultivation of *E. multilocularis* metacestodes in vitro. The co-culture system contains host support or feeder cells for the parasite to be maintained in medium for the several months required to produce PSC. Without the feeder cells, the metacestodes can be only maintained for a few weeks [14–16].

In the present study, we cultured PSC of *E. multilocularis* in a modified medium without host feeder cells. In the culture system, PSC re-differentiate into vesicles which can be maintained for more than 100 days and the fast growing vesicles produced PSC in brood capsules.

## Methods

### Animal infections and parasites

Mongolian jirds (*Meriones unguiculatus*) were used for maintaining larval *E. multilocularis* in an animal house at the Veterinary Research Institute, Xinjiang Academy of Animal Science. Jirds were intraperitoneally (*i.p.*) infected with PSC of *E. multilocularis* collected from Yili, Xinjiang, China. Pathogen-free female BALB/c mice, aged 6–8 weeks, were purchased from Beijing Vital River Laboratory Animal Technology Company Limited, and raised in the animal facility of the First Affiliated Hospital of Xinjiang Medical University.

### Collection of protoscoleces and in vitro cultivation

Mongolian jirds were infected with PSC of *E. multilocularis* for 4–8 months, and those with a heavy infection were sacrificed with CO_2_ anaesthesia. Metacestode masses were collected from the peritoneal cavity of the infected jirds under aseptic conditions and then washed with sterile phosphate-buffered saline (PBS). After removal of possible host tissues, the masses were minced into small tissue pieces. The parasite tissues were then passed through a sterilized sieve (100 mesh sieve) by grounding with a glass pestle. The passed through parasite tissues containing PSC were washed five times with PBS containing 100 U/ml penicillin and 100 μg/ml streptomycin (PBS-PS) by placing the tube on the bench for 5 min to allow the PSC to sediment at room temperature. The sedimented PSC were digested with 1 % (w/v) pepsin (Sigma-Aldrich, Louis, MO, USA) at pH 2.0 (adjusted with 2 M HCl) in Hank's Buffer for 20 min at 37 °C. After the digestion procedure, the PSC were washed five more times with PBS-PS to remove debris by natural sedimentation for 5 min at room temperature. The viability of PSC was determined by staining with 0.1 % methylene blue, with dead PSC staining blue.

Only samples with ≥ 95 % viability were subsequently cultured in RPMI 1640 medium (Gibco, Auckland, New Zealand) containing 25 % (v/v) FBS (Gibco), 0.45 % (w/v) yeast extract, 0.4 % (w/v) of glucose, 100 U/ml penicillin and 100 μg/ml streptomycin in a culture flask at 37 °C in the presence of 5 % CO_2_. The medium was changed every 3 days. Each culture was monitored weekly under an optical microscope to check the growth status of the larval vesicles.

### Animal infection

BALB/c mice were each *i.p.* transplanted with 30 small (250–300 μm in diameter) cultured vesicles of *E. multilocularis*. After 6 months, parasitic masses were collected from the mice and histopathological examination was performed by hematoxylin and eosin staining.

## Results and discussion

**Fig. 1:**
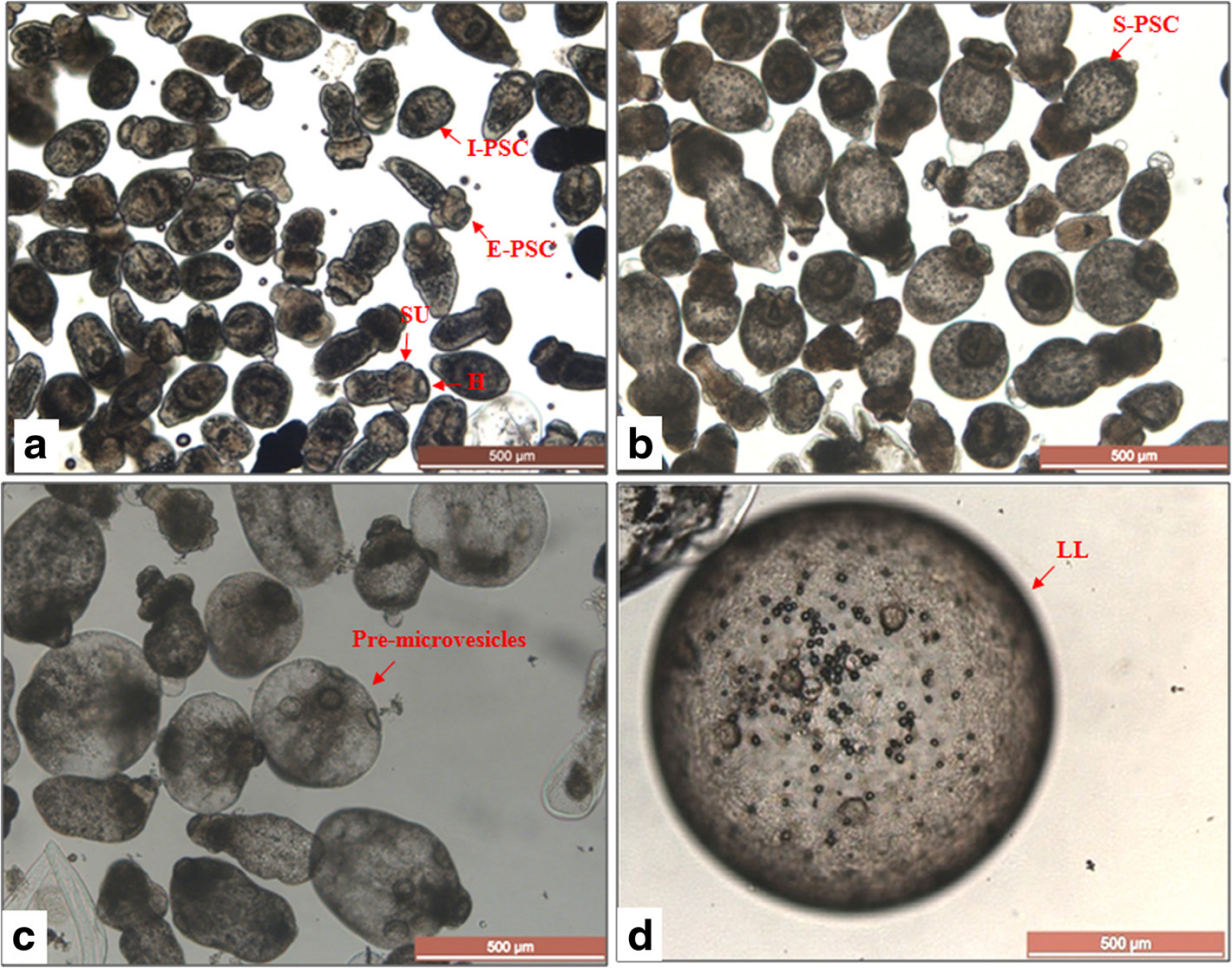
Light microscopy image of *E. multilocularis* vesicles obtained from protoscoleces cultured in vitro on day 1 (**a**), day 7 (**b**), day 15 (**c**), and day 60 (**d**). *Abbreviations*: E-PSC, evaginated PSC; H, hooks; LL, laminated layer; I-PSC, invaginated PSC; PSC, protoscoleces; SU, suckers; S-PSC, “swollen” PSC. *Scale-bars*: 500 μm

**Fig. 2:**
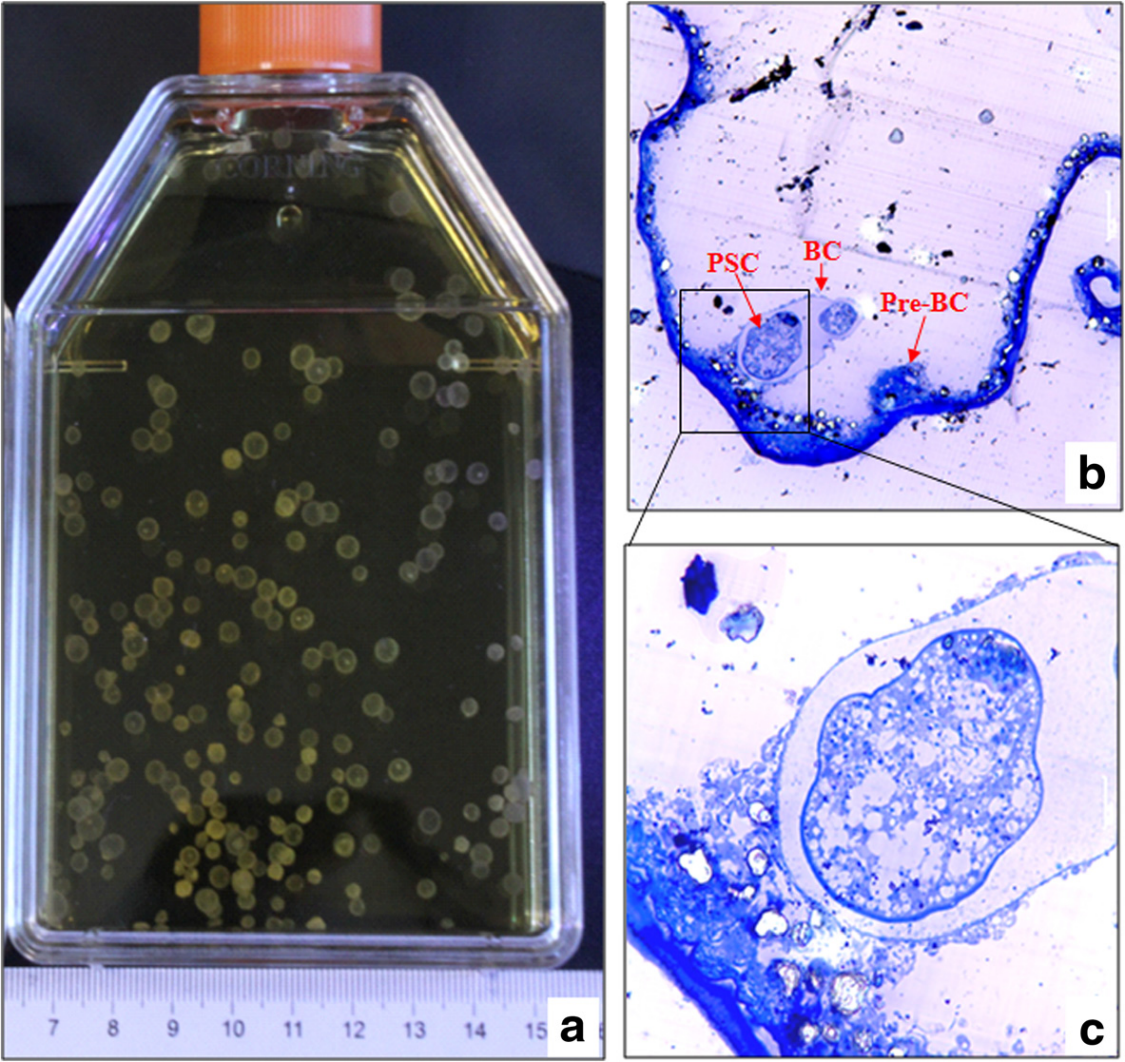
Protoscoleces cultured in vitro at day 100 (**a**) and toluidine blue staining of the vesicles (**b**, magnification: 100×; **c**, magnification: 400×). Vesicles obtained from in vitro culture showing a developed brood capsule containing mature and developing protoscoleces. *Abbreviations*: BC, brood capsule; Pre-BC, pre-brood capsule; PSC, protoscoleces

The genotype of the *E. multilocularis* Xinjiang Yili isolate used in this study was determined by sequencing the mitochondrial cytochrome *c* oxidase subunit 1 (*cox*1) gene. The sequence alignment showed 99.8 % identity to the sequence (gi261399263) of an *E. multilocularis* isolate collected in Kazakhstan (data not shown). On the first day of cultivation, we observed the culture contained 60 % of invaginated and 40 % evaginated PSC. The larval parasites had an even and smooth edge with clearly distinct internal and external structures/organs evident, including a rostellum, hooks, suckers and calcareous corpuscles (Fig. 1a). After 7 days of cultivation, a majority (76 %) of the PSC were evaginated. The head and neck region of the evaginated PSC were expanded into a “swollen” shape (Fig. 1b). After 15 days, the PSC were clearly differentiated into pre-microvesicles with some internal and external structures evident. However, the hooks and calcareous corpuscles had gradually disappeared or had degenerated. Suckers were re-positioned on the surface of the pre-microvesicles (arrowed in Fig. 1c). After 60 days, most PSC were dead, with only about 10 % of the PSC having developed into microvesicles. At this time, a thin laminated layer was observed in most vesicles under the microscope (Fig. 1d). The vesicles could be seen by the naked eye at this stage. As the dead PSC were normally sedimented, the microvesicles were suspended by slightly shaking the culture flask. Thus, the suspended microvesicles were easily separated from the dead PSC by simply transferring the suspended vesicles into a fresh flask with a Pasteur pipette. The culture system supported the healthy growth of the vesicles for more than 100 days, when the small vesicles grew to a mean diameter of 2.8 mm (Fig. 2a). Staining of the sectioned vesicles with toluidine blue indicated the small vesicles had a laminated layer; the germinal layer was also clearly stained. In part of the germinal layer, some cells were aggregated, likely forming pre-brood capsules. Importantly, PSC were found in some brood capsules and each capsule contained more than one PSC including immature and mature PSC (Fig. 2b, c).

**Fig. 3:**
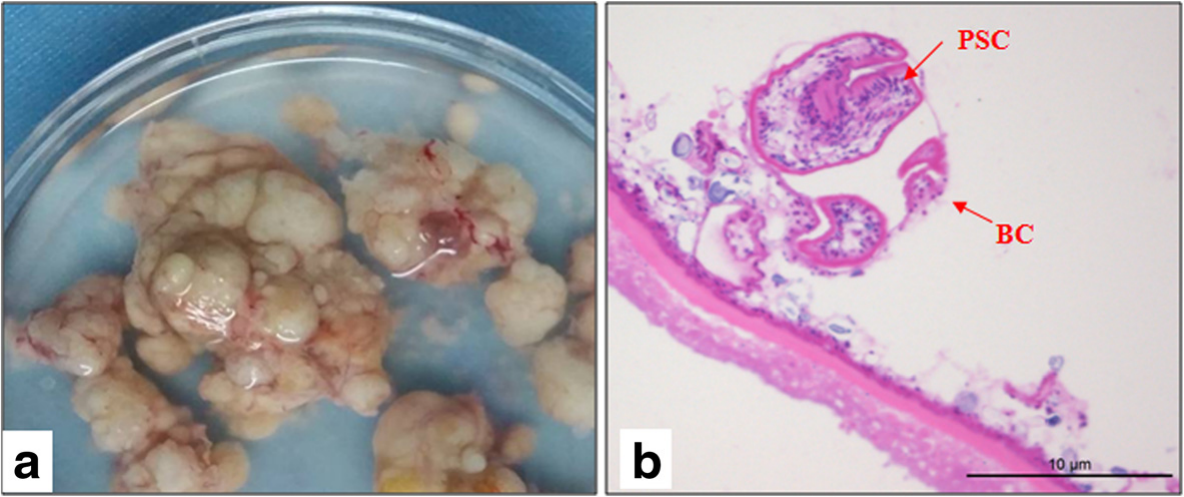
*Echinococcus multilocularis* metacestode and protoscolex development in mice. Cultured small vesicles were transferred into the peritoneal cavity of mice. **a** Metacestode masses of *E. multilocularis* in a mouse. **b** Hematoxylin and eosin staining of *E. multilocularis* lesion tissue section on a slide. *Abbreviations*: BC, brood capsule; PSC, protoscoleces. *Scale-bar*: 10 μm

In order to establish an in vivo animal model, we transferred 30 of the small, 300–500 μm in diameter sized cultured vesicles intraperitoneally into each of 10 female BALB/c mice (weight, 18–22 g) using a method described previously [17]. The mice were sacrificed 6 months post-infection. The subsequent lesion tissue/masses were removed from the peritoneal cavity of the mice for weighing and histological analysis. All the mice were successfully infected with the *E. multilocularis* tissue lesions forming many large tumor-like structures. The mean weight of the metacestode masses was 10.06 ± 3.58 g per mouse. Histological staining showed that the vesicles possessed the typical structure of a metacestode with a germinal membrane, a laminated layer, and brood capsules with mature and immature PSC (Fig. 3). This successful infection indicated that the in vitro cultured vesicles could be used as seeds for further infecting mice for maintaining the parasite in the laboratory.

Several studies have described the establishment of an in vitro cultivation system for *E. multilocularis* metacestodes. The first attempts were undertaken in the 1990s by Hemphill & Gottstein [14] and Jura et al. [15] using co-cultivation of host feeder cells and metacestode vesicles (fibroblasts in the “tissue block” method of Hemphill et al.; metacestodes with hepatocytes in the presence of a collagen matrix forming “sandwich configuration” method of Jura et al.). Subsequently, Spiliotis et al. (2004) [16] developed an in vitro system for a long-term cultivation of *E. multilocularis* larvae which depended strictly on reducing and anaerobic conditions. However, the growth of metacestode vesicles and differentiation towards the protoscolex stage only occurred in culture medium that was pre-conditioned by hepatoma cells. In 2008, Spiliotis & Brehm [18] established a large-scale cultivation system which utilized homogenized metacestode tissue that was incubated in liquid culture together with rat hepatoma feeder cells. The disadvantage of these culture methods was the requirement for the continuous presence of host cells. However, in this study, we have described a simple method which involves a medium containing a large amount of FBS (25 %) which allows PSC to redifferentiate into vesicles. The generated vesicles were able to produce PSC in vitro in the absence of host feeder cells, indicating that the high concentration of FBS likely contains sufficient essential growth factors for PSC growth and proliferation/differentiation. The advantage of this procedure and its comparison with other methods are described in Table 1. To obtain host cell-free cultured parasites, we used two procedures to remove possible host contamination: digestion with pepsin and washing. Before culture, we digested the parasite materials with pepsin and then washed the preparation five times with PBS. We then cultured the larval parasites in a feeder cell-free medium for more than 100 days. To determine whether the culture system required a high concentration of FBS, we used a low concentration of FBS (10 %) in the culture medium. We found PSC/microvesicles could not be maintained for more than six weeks, indicating that a high concentration of FBS is a key factor for PSC growth and differentiation. The success of culturing PSC to vesicles which in turn produced PSC indicates that larval *E. multilocularis* can be maintained in culture vessels in vitro. In addition, we showed that RPMI1640 is better than other media, such as MEM, for promoting PSC differentiation and maintenance [19].

**Table 1:** A comparison of different conditions used for culturing *E. multilocularis* in vitro

	Hemphill et al. [14]	Jura et al. [15]	Spiliotis et al. [16]	This study
Initial parasite material	Small tissue blocks/ vesicles	Homogenized parasite tissue	Small vesicles	protoscoleces
Adding feeder cells	Yes^a^	Yes^b^	Yes^c^	No
PSC formation	Yes	Yes	Yes	Yes
Culture time	Stopped at day 100	At least 2 months	Stopped at day 56	More than 100 days

^a^Culture medium: Initiated by first growing human cancer colon (CACO2) cells to confluency. Then RPMI 1640 containing 12 mM HEPES, 10 % FCS, 2 mM glutamine, penicillin (200 U/ml), streptomycin (200 μg/ml), fungizone (0.50 μg/ml) and 50 μM β-mercaptoethanol was added

^b^Culture medium: 10 % FBS, 9.6 mg prednisolone/ml, 0.014 mg glucagon/ml, 0.16 U insulin/ml, penicillin (200 U/ml) and streptomycin (200 μg/ml). Primary hepatocytes and parasitic tissues were both embedded in a collagen bilayer

^c^Culture medium: DMEM containing 100 U/ml penicillin G and 100 μg/ml streptomycin, 10 % FBS, different feeder cell lines, 0.01 % 2-mercaptoethanol, 100 μM L-cysteine and 10 μM bathocuproine disulfonic acid

## Conclusions

We have developed a simple and practical in vitro culture method for generating vesicles from PSC of *E. multilocularis* which in turn produced PSC in vitro in the absence of host feeder cells. This successful development of vesicles in a host feeder cell-free cultivation system represents a useful model for investigating the biology of *E. multilocularis* and for screening drugs for treatment of alveolar echinococcosis. Furthermore, we have developed a method for the secondary infection of *E. multilocularis* by simply transferring the cultured vesicles into the peritoneal cavity of mice, which provides a valuable animal model for investigating parasite development and elucidating host-parasite interactions.

## Abbreviations

AE, alveolar echinococcosis; ABZ, albendazole; BC, brood capsule; *cox*1, cytochrome *c* oxidase subunit 1; E-PSC, evaginated PSC; FBS, fetal bovine serum; H, hooks; *i.p,* intraperitoneally; I-PSC, invaginated PSC; LL, laminated layer; MBZ, mebendazole; PSC, protoscoleces; PBS, phosphate-buffered saline; Pre-BC, pre-brood capsule; S-PSC, “swollen” PSC; SU, suckers
